# Raman Spectroscopy: A Novel Technology for Gastric Cancer Diagnosis

**DOI:** 10.3389/fbioe.2022.856591

**Published:** 2022-03-15

**Authors:** Kunxiang Liu, Qi Zhao, Bei Li, Xia Zhao

**Affiliations:** ^1^ State Key Laboratory of Oncology in South China, Collaborative Innovation Center for Cancer Medicine, Department of Oncology, Sun Yat-sen University Cancer Center, Guangzhou, China; ^2^ State Key Laboratory of Applied Optics, Changchun Institute of Optics, Fine Mechanics and Physics, Chinese Academy of Sciences, Changchun, China; ^3^ University of Chinese Academy of Sciences, Beijing, China; ^4^ Cancer Microbiome Platform, Sun Yat-sen University Cancer Center, State Key Laboratory of Oncology in South China, Collaborative Innovation Center for Cancer Medicine, Guangzhou, China; ^5^ Department of Microbiology, Army Medical University, Chongqing, China

**Keywords:** Raman spectroscopy, gastric cancer, clinical diagnostics, on-site applications, machine learning

## Abstract

Gastric cancer is usually diagnosed at late stage and has a high mortality rate, whereas early detection of gastric cancer could bring a better prognosis. Conventional gastric cancer diagnostic methods suffer from long diagnostic times, severe trauma, and a high rate of misdiagnosis and rely heavily on doctors’ subjective experience. Raman spectroscopy is a label-free molecular vibrational spectroscopy technique that identifies the molecular fingerprint of various samples based on the inelastic scattering of monochromatic light. Because of its advantages of non-destructive, rapid, and accurate detection, Raman spectroscopy has been widely studied for benign and malignant tumor differentiation, tumor subtype classification, and section pathology diagnosis. This paper reviews the applications of Raman spectroscopy for the *in vivo* and *in vitro* diagnosis of gastric cancer, methodology related to the spectroscopy data analysis, and presents the limitations of the technique.

## Introduction

Gastric cancer is the fifth most commonly diagnosed cancer worldwide and the third most common cause of cancer-related death because it is usually advanced at the time of diagnosis (Bray et al., 2018). The development of gastric cancer is a multistep process, including chronic non-atrophic gastritis, multifocal atrophic gastritis, intestinal metaplasia, dysplasia, and invasive carcinoma (Correa and Piazuelo, 2008). The prognosis of gastric cancer is closely related to the disease course, and after effective treatment, the 5-year survival rate of early gastric cancer reaches more than 90% (Nallala et al., 2012). Early detection is the most important factor to improve gastric cancer survival and prognosis. Thus, efficient and sensitive examination techniques are critical for increasing early detection rates.

Endoscopic diagnosis, pathological diagnosis, and medical images are commonly used in clinical gastric cancer diagnosis, and these diagnostic techniques have the disadvantages of long diagnosis times, serious trauma, and a high rate of misjudgment, and rely heavily on doctors’ subjective experience. As a result, a new gastric cancer diagnosis technique that is fast, non-invasive, highly accurate, and sensitive is desperately needed. Raman spectroscopy, a molecular vibrational spectroscopy technique, can identify the properties and structures of various substances by obtaining fingerprinting information from molecules. Raman spectroscopy is currently used to diagnose a variety of tumors.

Kim H. H. reviewed the application of fiber optic Raman in the *in vivo* study of gastric cancer, but it was limited to *in vivo* study (Kim, 2015). In addition to this, Auner G. W. et al. summarized the applications of Raman spectroscopy in cancer diagnosis (Auner et al., 2018), and Baker M. J. et al. reviewed Raman spectroscopy in clinical applications (Baker et al., 2018). This paper focuses on the field of gastric cancer based on a review of 35 articles found using the keyword “gastric cancer Raman” and reviews the research progress of Raman spectroscopy in the early diagnosis of gastric cancer. This paper not only reviews the research related to Raman spectroscopy for the *in vivo* and *vitro* diagnosis of gastric cancer and its future prospects in the clinical field, but also focuses on Raman spectroscopy data analysis methods used in these applications.

## Raman Spectroscopy and Tumor Diagnosis

**FIGURE 1:**
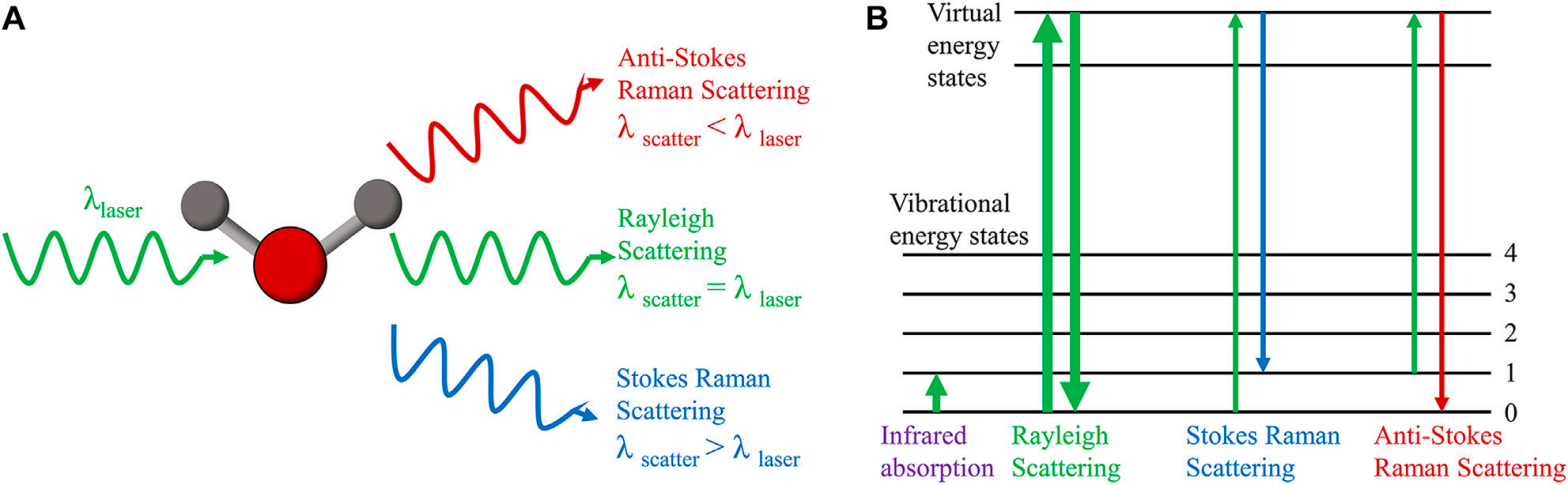
Principle of Raman scattering. **(A)** Raman scattering and Rayleigh scattering. **(B)** Energy level diagram of Raman scattering, Rayleigh scattering and infrared absorption.

In 1928, Indian scholar C. V. Raman discovered that when monochromatic light of a fixed frequency is incident on a medium, two scattering processes occur simultaneously: one is Rayleigh scattering with constant frequency, which is caused by elastic collisions between incident photons and matter, and the other is frequency-changing Raman scattering, which is caused by an energy exchange that occurs when incident photons collide with matter, causing a change in the molecules’ vibrational or rotational energy levels (Raman and Krishnan, 1928). In Raman scattering, the scattering in which a portion of the energy of the photon is transferred to the molecule is called Stokes Raman scattering, while the scattering in which the energy of the vibration and rotation of the molecule is transferred to the photon is anti-Stokes Raman scattering (Figure 1). The Raman shift, which is the transverse coordinate of the Raman spectrum that we see, is the difference between the frequency of the scattered light and the frequency of the incident light (Rayleigh scattering) during Raman scattering (Bergholt et al., 2014). The Raman shift is determined by the molecule’s vibrational energy level, which varies depending on the chemical group, regardless of the excitation wavelength. The intensity of the Raman peak, which is related to the excitation light wavelength, power, concentration, and molecular polarization rate of the substance being tested, as well as the instrument system’s collection efficiency, is the vertical coordinate of the Raman spectrum. All molecules, including gas, solid, and liquid molecules, have their own Raman spectra. As a result, Raman spectroscopy can determine the properties and structure of various substances without the need for sample labeling.

The excitation light source, focusing system, filter, dispersion system, detector, and recording system are all components of a Raman spectrometer. Spontaneous Raman spectroscopy is a name given to distinguish other Raman scattering. Raman signals have low intensity and poor interference resistance, necessitating a long integration time in practical applications to obtain a spectrum with an acceptable signal-to-noise ratio, which is inconvenient for spectral acquisition and fast imaging. Furthermore, the Raman signal can be affected by the fluorescence background in biological samples. Various Raman signal enhancement techniques have emerged to address the challenges of slow acquisition speed and low signal-to-noise ratio. Resonance Raman spectroscopy (RRS) (Liu et al., 2020; Zhao and Mu, 2020), surface-enhanced Raman spectroscopy (SERS) (Kneipp et al., 1997; Lee and Meisel, 2002; Qian et al., 2008), tip-enhanced Raman spectroscopy (TERS) (Chen C. et al., 2014) and stimulated Raman spectroscopy (SRS) (Mittal et al., 2013) are the most commonly used Raman signal enhancement techniques. All of these techniques have high biochemical sensitivity and selectivity, and none of these techniques require the sample to be labeled, making them promising in biological and medical fields. Due to the complexity of the environment and the characteristics of the Raman spectroscopy signal, some overlapping peaks are inevitable in the signal acquisition process. It has been demonstrated that overlapped spectral peaks can be identified by combined symmetric zero-area conversion and L-M fitting (Bi et al., 2015). The evolving spectral pre-processing and classification algorithms greatly compensate for the influence of the external environment and spectrometer on the quality of the spectra, with high specificity and accuracy in numerous applications.

In the application of tumor diagnosis, researchers collect Raman spectroscopy data from samples of tumor tissues, cancer cells, body fluids or tumor markers and use data processing methods such as statistics, machine learning, and deep learning to achieve the diagnosis of different tumors (Ralbovsky and Lednev, 2020). Raman spectroscopy is currently being used to diagnose malignant tumors in various parts of the human body, including the bladder (Bovenkamp et al., 2018), breast (Haka et al., 2009), brain (Mizuno et al., 1994), cervix (Kamemoto et al., 2010), colorectum (Lin et al., 2011), esophagus (Feng et al., 2013), gastrointestinal tract (Teh et al., 2008a), liver (Xiong et al., 2012), lung (Ye et al., 2014), and oral cavity (Cals et al., 2015), as well as in the diagnosis of nasopharyngeal carcinoma (Lin K. et al., 2017), laryngeal carcinoma (Lin et al., 2016b), and ovarian cancer (Baker et al., 2018).

## Raman Spectroscopy for the Diagnosis of Gastric Cancer

Endoscopic diagnosis, histopathology, clinical imaging, tumor markers, and other clinical methods are currently used to detect gastric cancer. The “gold standard” in clinical tumor diagnosis is endoscopic and histopathological diagnosis, which is performed on a tissue and cellular scale. After staining the tissues, by observing the size, density and nucleoplasmic ratio of the cells under the microscope, the structure, number and arrangement of the glands, it is possible to determine the tissue type of normal gastric mucosa, precancerous lesions and gastric cancer. Its detection time is about 3 days. But this necessitates biopsy sampling for tissue diagnosis, which can result in gastrointestinal bleeding and perforation. Furthermore, pathological diagnosis accuracy is influenced to some extent by pathologists’ subjective factors, and test procedures are time consuming and results can be delayed. X-ray contrast radiography examination, computed tomography examination, magnetic resonance imaging (MRI) and other imaging diagnostic methods are commonly used as clinical diagnostic tools, but they can only make preliminary judgments about the shape and nature of pathological tissues. Because they can only make a preliminary assessment of the shape and nature of pathological tissues, and because their spatial resolution is low, these techniques have a low detection rate for microscopic tumors and early gastric cancer. The commonly used clinical markers are CEA, CA19-9, CA125, and CA72-4, etc. The use of tumor markers for gastric cancer diagnosis is susceptible to individual differences and some benign diseases, and their low specificity and sensitivity limit their application in gastric cancer diagnosis. Its detection time takes a few days. Its detection time takes a few days. More importantly, tumor markers are very difficult to detect. This emphasizes the value of Raman spectroscopy in the early detection of gastric cancer.

The composition and content of chemical substances in stomach tissues or cells will change to some extent during the cancerous process and can be directly reflected by the corresponding characteristic peaks and intensity of the Raman spectra. The comprehensive sensitivity and specificity of Raman spectroscopy for the diagnosis of gastric cancer are 0.89 and 0.92, respectively, when histopathology is used as the reference standard. As a result, Raman spectroscopy is an objective and sensitive optical diagnostic technique for detecting gastric cancer that is non-invasive to the body, allows for real-time diagnosis, and is simple to use (Ouyang et al., 2015).

**FIGURE 2:**
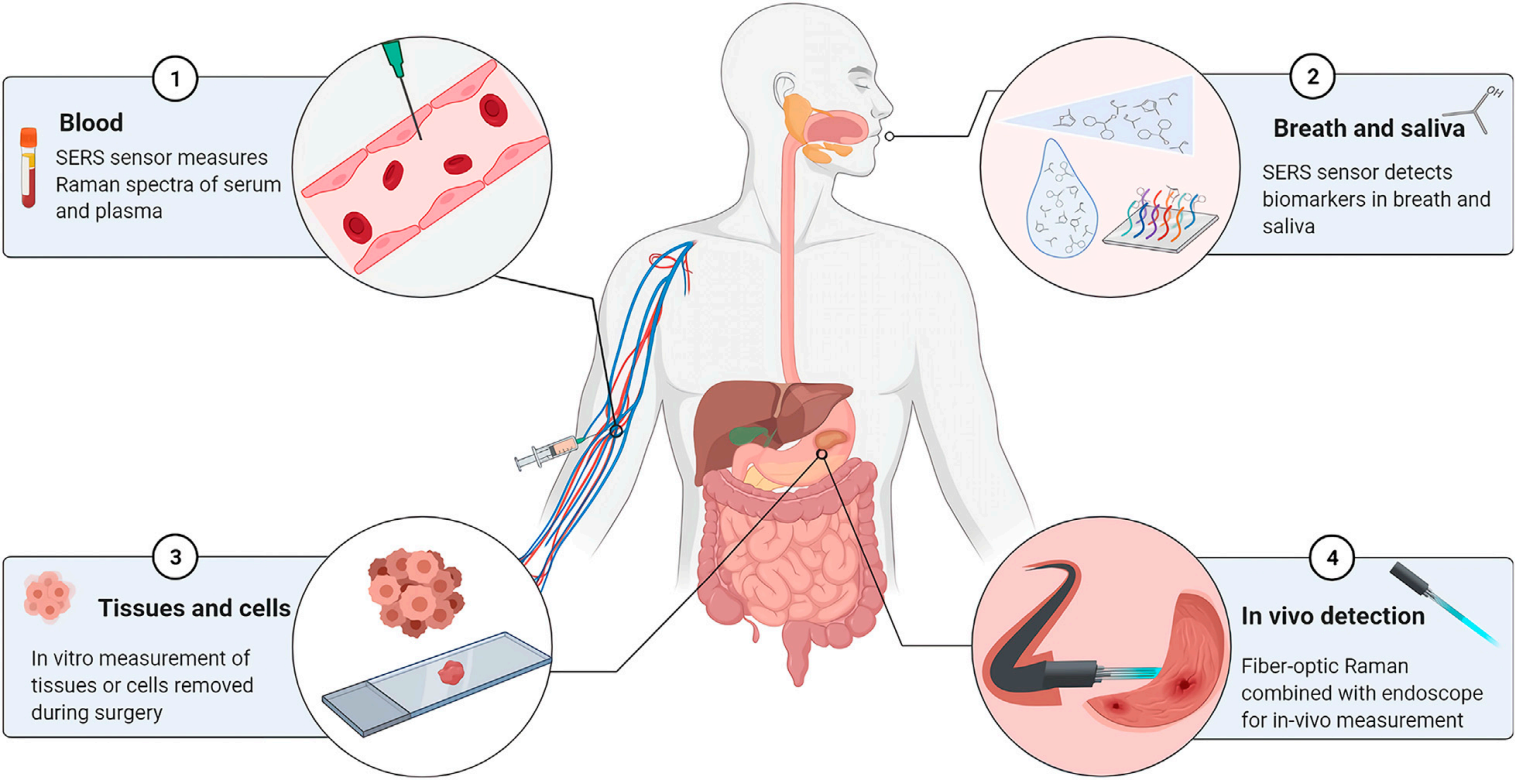
Sample types for Raman spectroscopy in gastric cancer diagnostic studies. ① Blood samples. Diagnostic analysis of serum, serum protein, serum RNA, or plasma by SERS sensor. ② Breath and saliva. SERS coupled with mass spectrometry to detect biomarkers in the sample. ③ Tissue or cell. Detection of isolated tissue samples by spontaneous Raman or confocal Raman spectroscopy. ④ *In vivo* detection. Raman *in vivo* measurements using fiber optic Raman in combination with endoscopy.

The current samples in Raman-related studies of gastric cancer are primarily derived from tissues excised during surgery, isolated cell samples, body fluids, or endoscopic biopsies, as shown in Figure 2. To analyze tissue or cell samples *in vitro*, spectral data are collected primarily using spontaneous Raman spectroscopy with laser wavelengths of 532 nm, 785 nm, or 1,064 nm. In addition, some studies have used confocal Raman spectroscopy techniques (Shen et al., 2005; Hu et al., 2008), which has the advantage of having a high spatial resolution that effectively eliminates signal interference from layers beyond the focal plane. SERS was primarily used to study samples such as blood, breath, and saliva, with a maximum enhancement of 10^15^ times compared to normal Raman spectra of molecules (Hung et al., 2010). Furthermore, fiber optic Raman spectroscopy plays an important role in the real-time detection and *in situ* analysis of gastric cancer patients using fiber optic probes *in vivo*.

### *In Vitro* Diagnosis of Gastric Cancer by Raman Spectroscopy

Raman spectroscopy of *ex vivo* samples was collected and analyzed to identify and classify chemical differences among different samples.

#### Raman Spectroscopy of Gastric Tissue and Cell Samples *In Vitro*

Raman spectroscopy data acquisition and classification of gastric tissues or cells is the most direct and widespread application of Raman spectroscopy in diagnosing the nature of gastric tumors. Traditional intraoperative histological diagnosis requires workflows, such as tissue transport, sample processing, section preparation and tissue diagnosis, while the Raman spectroscopy-based gastric cancer diagnosis method is able to collect data directly from fresh tissues.

Formalin-fixed paraffin-embedded (FFPE) samples (Ikeda et al., 2018), frozen samples (Hu et al., 2008; Kawabata et al., 2008; Luo et al., 2013; Zhou et al., 2016), and fresh tissue samples (Kalyan Kumar et al., 2007; Teh et al., 2008a; Teh et al., 2008b; Teh et al., 2010a; Bergholt et al., 2011a; Luo et al., 2013; Chen Y. et al., 2014) are the most common samples used in Raman studies of isolated gastric tissues and cells. When the diagnostic results are known, FFPE samples can be studied, but the fixation of the tissue may affect its biochemical composition, resulting in changes in Raman peaks (Ramos et al., 2015). Frozen tissues are closer to the fresh state, and their Raman spectra have a good signal-to-noise ratio and reproducibility (Haka et al., 2009; Kamemoto et al., 2010). However, the freezing and thawing process appears to change the intensity of some Raman peaks, making detection less accurate than fresh tissues (Wills et al., 2009).

A desktop Raman spectroscopy system is typically used for Raman spectroscopy of isolated tissues. NIR light sources, particularly 785 nm lasers located in the high transmittance “optical window” of biological tissues, are frequently used in medical Raman measurement systems due to the relatively low absorption coefficient of NIR light by molecules in biological tissues that primarily absorb light (e.g., melanin, water, and lipids) (Pence and Mahadevan-Jansen, 2016). Visible light with a wavelength of 532 nm has a higher scattering intensity than NIR light (Ikeda et al., 2018), but NIR light with wavelengths of 785 nm (Kalyan Kumar et al., 2007; Teh et al., 2008a; Teh et al., 2008b; Teh et al., 2010a; Teh et al., 2010b; Bergholt et al., 2011a; Luo et al., 2013; Chen Y. et al., 2014; Zhou et al., 2016) and 1,064 nm (Kawabata et al., 2008) is effective in reducing tissue autofluorescence and has been used more often in diagnostic studies of gastric cancer.

Through the transmission system, light is irradiated onto the sample, and the Raman signal generated by the tissue sample is collected and processed by the detector and spectrometer. Raman spectra are typically acquired in the range of 400–3,500 cm^−1^, and this wavenumber region can be divided into three parts: the Raman fingerprint region (FP, 400–1800 cm^−1^), the high wavenumber region (HW, 2,800–3,200 cm^−1^), and the intermediate region, which typically lacks Raman characteristic peaks. Because the acquisition range of the spectra affects the acquisition time and redundancy of subsequent data processing, most studies only acquire spectral data in the FP region or both the FP and HW regions. According to related research, analyzing the FP region alone is superior to analyzing the HW region alone (Zhou et al., 2016), and analyzing the FP and HW regions simultaneously is superior to analyzing the FP and HW regions separately (Lin et al., 2016a). Furthermore, studies have shown that increasing the laser power has little effect on overall prediction accuracy, but increasing the integration time can significantly improve the prediction accuracy (Chen et al., 2019). The choice of different spectral ranges and spectral acquisition conditions can affect the diagnostic results, so the desired experimental conditions should be chosen based on the sample characteristics in the experiment.

As a fast and non-invasive method of detecting gastric tissues, Raman spectroscopy has made significant progress. Raman detection of isolated gastric tissues has been shown in other studies to diagnose gastric dysplasia (Teh et al., 2008b), differentiate gastric adenocarcinoma staging (Teh et al., 2010a), identify malignant gastric mucosal tissues (Kalyan Kumar et al., 2007), differentiate precancerous and cancerous tissues (Luo et al., 2013), and diagnose gastric cancer (Ikeda et al., 2018). In addition, Chen Y. et al. (2014). looked at the differences in the characteristic peak spectra of genomic DNA, nuclei, and tissues of normal and malignant gastric mucosa to see how they changed the spatial structure and biochemical composition of mucosal tissue during carcinogenesis. Teh et al. (2010b) also showed that NIR Raman spectroscopy can be used to diagnose *Helicobacter-pylori* infection and inflammatory damage in the stomach at the molecular level.

**FIGURE 3:**
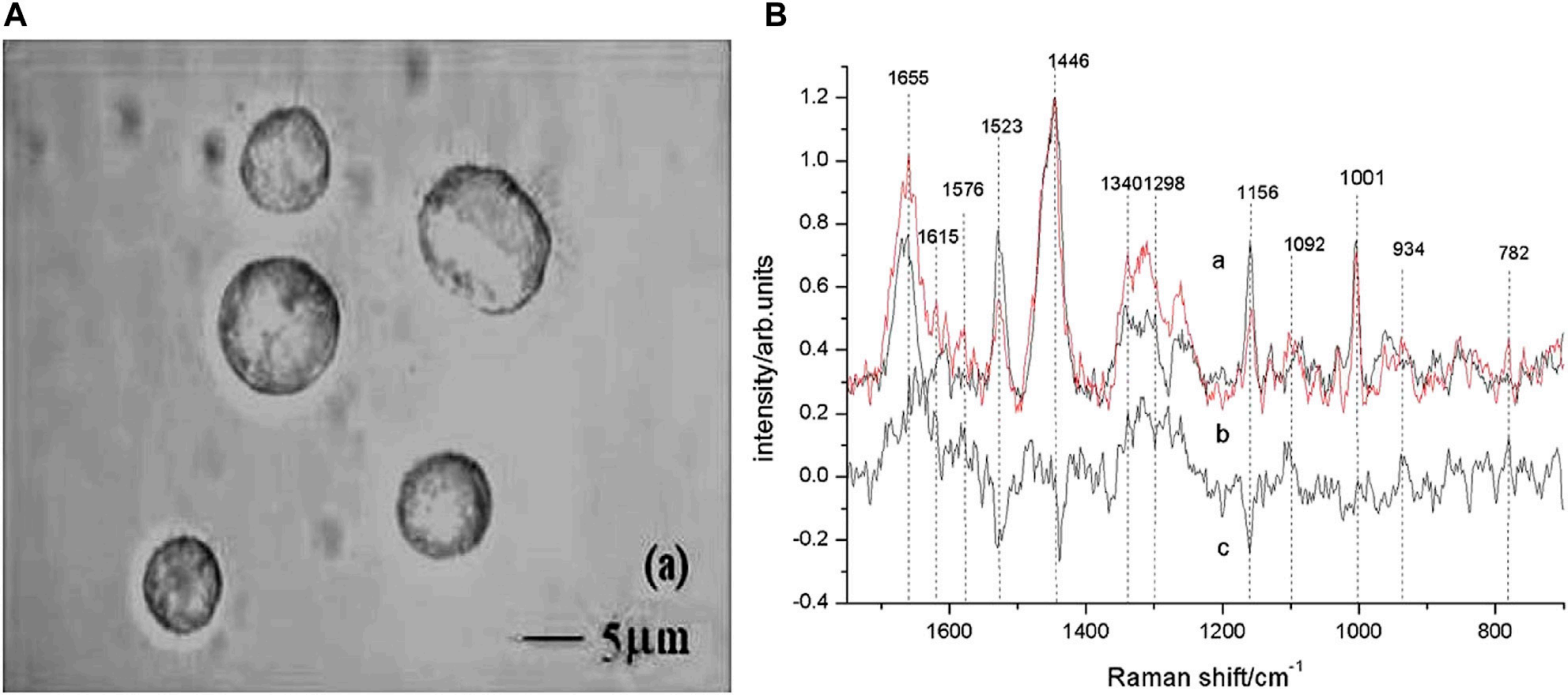
Micrographs of gastric cancer cells with Raman spectra. **(A)** Images of gastric carcinoma cells observed by differential interference contrast (DIC) microscope with the ×100 objective lens. **(B)** Raman spectra of untreated gastric carcinoma cells (curve a) and apoptotic cells (curve b). Curve c was the difference spectrum between a and b. The position of Raman bands at 782, 934, 1,001, 1,092, 1,156, 1,298, 1,340, 1,446, 1,523, 1,576, 1,615, and 1,655 cm^−1^ were marked. Reproduced from permission (Yao et al., 2009). Copyright (2009), with permission from Elsevier.

Cells are more refined than tissues on the detection scale. Based on Raman spectroscopy of cultured single cells, Shen et al. (2005) successfully screened suspected gastric mucosal malignant cells in 3 min. The main compositional differences between apoptosis-inducing cells and gastric cancer cells were discovered by Yao et al. (2009), allowing non-destructive Raman spectroscopy to be used to monitor apoptosis in gastric cancer cells and to test new drugs against the therapeutic effects of various cancer cells. The microscopic pictures of gastric cancer cells with Raman spectra are shown in Figure 3.

#### Raman Spectroscopy for Body Fluid Samples *In Vitro*

Blood components, such as plasma, red blood cells, white blood cells, and platelets, are important body fluids used to monitor numerous disease states. Raman spectroscopy has been used to detect blood components and whole blood for more than 40 years (Atkins et al., 2017). Since the individual components of cancerous blood undergo small changes that are difficult to detect by normal methods, the SERS technique, which is more sensitive, is mostly used in research to detect them.

**FIGURE 4:**
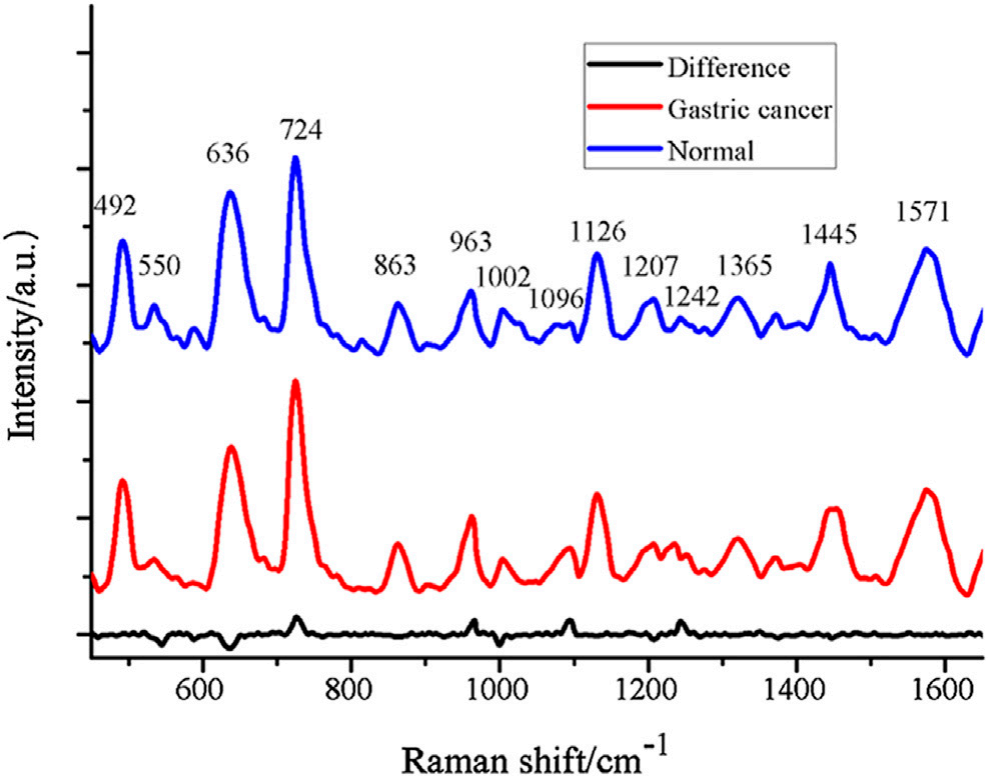
The contrast of SERS spectrum of gastric cancer and normal with Au/SiNWA substrate. Reproduced from permission (Wei et al., 2016). Copyright (2016), with permission from Elsevier.

Because molecules show unusually large Raman scattering cross-sections when adsorbed on metal surfaces at a certain nanometer size, the key to SERS is the preparation of enhanced substrates or nanoenhanced materials. Gold and silver nanomaterials are commonly used in SERS studies, and related studies have shown that 3D nanostructured gold nanoparticle/silicon nanowire array (Au/SiNWA) SERS substrates are also a good way to distinguish between gastric and normal serum SERS-active substrates (Wei et al., 2016). The Raman spectra of Au/SiNWA substrate compared with the SERS spectrum of normal gastric cancer are shown in Figure 4. Furthermore, plasma SERS spectra are affected by different laser polarizations (Feng et al., 2011a). According to a number of studies, using SRES to detect plasma and serum samples can not only identify gastric cancer (Feng et al., 2011b; Chen et al., 2012; Guo et al., 2019; Avram et al., 2020), but also differentiate between different stages of gastrointestinal tumors and different digestive system cancers (Lin J. et al., 2017).

Specific odors in the breath were linked to certain diseases by ancient Greek physicians (Kim et al., 2012). Since 1971, studies have shown breath to contain a complex mix of nearly 3,000 volatile organic compounds (VOCs) (Peng et al., 2010; Amal et al., 2012; Hakim et al., 2012). Cancer cells or tissues that are subjected to metabolic or oxidative stress produce specific VOCs. As a result, they can be used as VOC biomarkers for cancer without affecting clinical symptoms (Konvalina and Haick, 2014). Furthermore, saliva can directly reflect a person’s health status and has obvious benefits for use in the diagnosis of gastric cancer. Studies have shown that using gas chromatography–mass spectrometry coupled with solid phase microextraction (SPME) and SERS to identify organic compound biomarkers in breath or saliva can identify early and advanced gastric cancer (Chen et al., 2016; Chen et al., 2018). Based on the SERS sensor, this non-invasive, rapid, and reliable analysis method offers a new strategy for identifying gastric cancer patients in the general population.

### *In Vivo* Diagnosis of Gastric Cancer by Raman Spectroscopy

**FIGURE 5:**
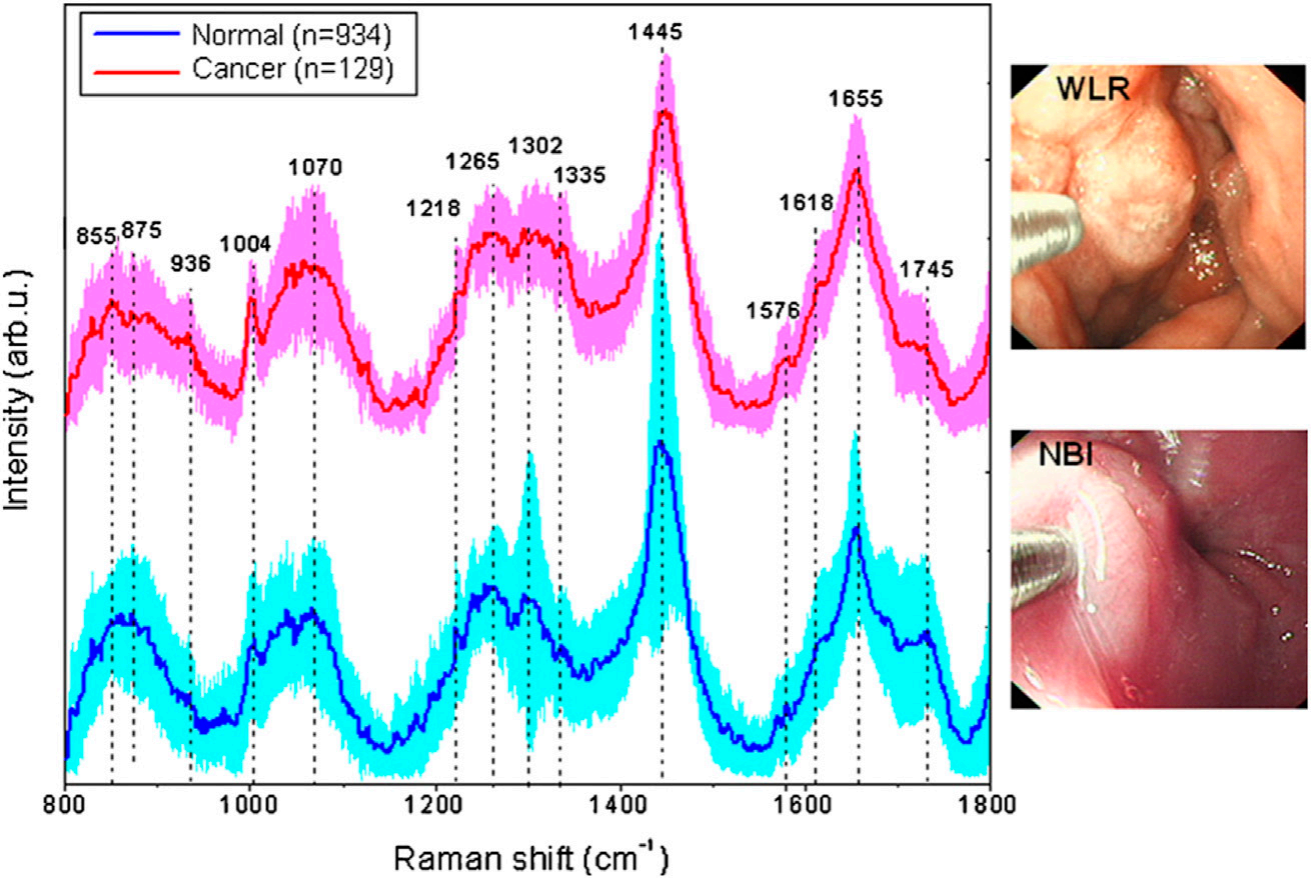
*In vivo* mean Raman spectra ±1 standard deviations (SD) of normal (*n* = 934), and cancer (*n* = 129) gastric tissue, as well as the corresponding white-light reflectance (WLR) image and narrow-band image (NBI) acquired during clinical gastroscopic examination. Reproduced from permission (Huang et al., 2010b). Copyright (2010), with permission from Elsevier.

Many researchers want to bring Raman spectroscopy technology from the lab to *in situ* testing in the field, and fiber optic Raman technology allows for *in situ* testing and biomedical *in vivo* testing in the field. The fiber optic Raman probe, which is made up of an optical fiber and a small lens, can detect any part of a sample to obtain the Raman spectral information for the sample detection site. The use of fiber optic Raman combined with endoscopy in the diagnosis of gastric cancer allows not only the acquisition of real-time images of the tumor, but also simultaneous Raman spectral data acquisition for non-destructive and flexible detection of the digestive tract (Duraipandian et al., 2012; Wang et al., 2015). Raman spectra and gastric cancer images obtained using an image-guided Raman endoscopy system are shown in Figure 5. Before using fiber-optic Raman to acquire Raman spectra *in vivo*, it is necessary to first observe the gastric mucosa and the gastric lumen for any adhesions or fluids through the gastroscope. After rinsing and aspirating away the adherent, the location of the target Raman acquisition site can be determined with the aid of endoscopy to exclude interference from other tissues and blood vessels.

Quartz-based optical fibers, which have incomparable advantages in terms of light transmission, *in vivo* compatibility, and bending flexibility, are currently used for Raman spectroscopy detection of biological tissues. The structural design of the fiber optic probe is determined by the application; for example, for *in vivo* detection of the stomach, the probe must be smaller and more flexible. Furthermore, a previous study found that changing the tissue pressure and angle of the probe had no significant effect on the Raman spectrum (Shim et al., 2000).

A previous study using a small endoscopic system equipped with a miniature Raman probe demonstrated the safety of Raman detection in rats before it was used in human experiments (Hattori et al., 2007). Numerous studies have shown that the use of endoscopic Raman probes can diagnose gastric dysplasia (Huang et al., 2009; Huang et al., 2010a), benign and malignant ulcerative lesions of the stomach (Bergholt et al., 2010), and gastric cancer (Huang et al., 2010b). Otherwise, the probes can be used *in vivo* to collect the Raman spectra from different anatomical locations in the upper gastrointestinal tract (Bergholt et al., 2011a). Additionally, the combination of Raman spectroscopy and near-infrared autofluorescence can improve the accuracy of gastric cancer diagnosis (Bergholt et al., 2011b).

## Data Processing and Analysis of Raman Spectroscopy

Data preprocessing, feature selection, and multivariate modeling are all part of a typical spectral analysis workflow in Raman spectroscopy (Allegrini and Olivieri, 2013). Table 1 lists the analytical methods used in the Raman studies of gastric cancer discussed in the text.

**TABLE 1:** Raman spectroscopy chemometric analysis methods employed in gastric cancer research.

*In Vivo* or *Vitro*	Sample type	Objective	Preprocessed	Feature extraction	Classification method	Source
*in vitro*	Tissue	Identification of gastric cancer and normal tissue	—	—	—	Ikeda et al. (2018)
			SG, airPLS, Normalized	—	PLS-DA	Zhou et al. (2016)
			—	PCA	CART, LDA	Seng et al. (2008); Teh et al. (2008a)
		Identification of gastric dysplasia and normal tissue	MF	PCA	LDA	Teh et al. (2008b)
		Differentiating normal from different subtypes of gastric adenocarcinoma tissues	SG, BE, Normalized	PCA	MNLR	Teh et al. (2010a)
		Identification of normal and malignant gastric mucosal tissue	BE, Normalized	—	—	Hu et al. (2008)
			BC, MF, Normalized	PCA	Mahalanobis distance etc.	Kalyan Kumar et al. (2007)
		Comparative analysis of genomic DNA, nuclear, and tissue biochemical components between normal gastric mucosa and gastric cancer	BE, Normalized	Feature search	—	Chen Y. et al. (2014)
		Differentiating tumor from non-tumor tissue in patients with gastric cancer	—	PCA	—	Kawabata et al. (2008)
		Differentiating precancerous lesions from cancerous tissue from normal gastric tissue	airPLS, Normalized	PCA	LDA, NBC	Luo et al. (2013)
		Identification of *Helicobacter-pylori* infection and intestinal metaplasia	SG, BE	PCA	LDA	Teh et al. (2010b)
	Cells	Distinguishing gastric cancer cells in malignant gastric mucosa	Normalized	—	—	Shen et al. (2005)
		Analysis of gastric cancer cell apoptosis	BE, MF, BC, Normalized	PCA	—	Yao et al. (2009)
	Blood	Identification of gastric cancer and normal plasma	BE, Normalized	PCA	LDA	Feng et al. (2011b)
			BE, Normalized	PCA	LDA	Feng et al. (2011a)
		Differentiating serum proteins between normal and three digestive cancers	BE, Normalized	PCA	LDA	Lin J. et al. (2017)
		Identification of serum from normal and gastric cancer	BE, Normalized	—	—	Wei et al. (2016)
			—	PCA	CRM	Guo et al. (2019)
		Differentiating serum RNA from normal and gastric cancer	Normalized	PCA	LDA	Chen et al. (2012)
		Differentiating serum from normal and gastrointestinal tumors	—	PCA	QDA	Avram et al. (2020)
	Saliva	Identification of gastric cancer markers in saliva	—	PCA	LogReg	Chen et al. (2018)
	Breath	Identification of gastric cancer markers in breath	—	PCA	—	Chen et al. (2016)
*in vivo*	Tissue	Identification of gastric cancer and normal tissue	SG	PCA	LDA	Bergholt et al. (2011b)
			SG, BE, Normalized	ACO	LDA	Bergholt et al. (2011c)
			SG, BE, Normalized	PCA	PLS-DA	Duraipandian et al. (2012)
			Normalized	—	CART	Huang et al. (2010b)
		Identification of gastric dysplasia and normal tissue	—	—	PLS-DA	Wang et al. (2015)
			SG, BE, Normalized	PCA	LDA	Huang et al. (2010a)
		Identification of different gastric tissue cancers vs. normal	SG, Normalized	—	PLS-DA	Bergholt et al. (2011a)
		Identification of gastric intestinal metaplasia and normal tissues	SG, BE, Normalized	PCA	LDA	Lin et al. (2016a)
		Identification of benign and malignant gastric ulcer tissues	SG, BE	—	PLS-DA	Bergholt et al. (2010)
		Gastrointestinal tissue classification	BE, Normalized	PCA	ANN	Shim et al. (2000)

Notes: ‘-’ means that no such method was used or is not mentioned in the literature. Abbreviations: SG, Savitsky–Golay filter; airPLS, adaptive iteratively reweighted penalized least squares; PLS-DA, Partial least squares-discriminant analysis; PCA, Principal component analysis; LDA, linear discriminant analysis; CART, classification and regression tree; MF, mean filter; BE, background elimination; MNLR, multinomial logistic regression; BC, baseline correction; NBC, naive bayes classifier; CRM, characteristic ratio method; QDA, quadratic discriminant analysis; LogReg, Logistic regression; ACO, ant colony optimization; ANN, artificial neural network.

The spectral signal is preprocessed to remove unwanted variations and artifacts. Noise removal, baseline correction, scattering correction, and scaling are all common preprocessing techniques. Furthermore, spectral normalization (e.g., spectral area, maximum value, average intensity normalization, etc.) can help compare spectral shapes and relative peak intensities between different tissues by reducing the effects of fluctuations in the excitation intensity (Chen et al., 2013). Savitzky–Golay (SG) filtering is the most commonly used filtering method in studies applying Raman spectroscopy to gastric cancer (Table 1). The SG filter is a filtering method based on local polynomial least square fitting in the time domain, and its most significant characteristic is that it can ensure that the shape and width of the signal remain unchanged while filtering out noise.

The reason for feature selection is that spectral data contain a significant amount of invalid information, and analyzing the entire spectrum directly may affect the diagnostic effect while also increasing the computational cost. Algorithms are frequently used to perform feature selection and spectral extraction of the raw spectra, in addition to manually truncating the spectrum or selecting special peaks based on a priori knowledge. Principal component analysis (PCA) is the most commonly used feature extraction method in gastric cancer Raman studies (Table 1). The PCA algorithm is a popular unsupervised algorithm for reducing the difficulty of spectral data analysis before it is fed into multivariate models. However, reducing variables may result in the loss of useful information and a change in the spectral data’s original pattern.

In order to obtain the class information of the sample to be measured from the Raman spectral data, a mathematical relationship between the spectrum and the label needs to be established. Thus multivariate modeling is used to realize the identification and diagnosis of different samples. The most straightforward classification method is Raman peak intensity comparison (Shen et al., 2005; Hu et al., 2008; Chen Y. et al., 2014; Wei et al., 2016; Ikeda et al., 2018), but the accuracy is not high. The ant colony optimization algorithm (ACO) method of finding the best Raman features and the analysis method of discriminating peak feature intensities based on the Mahalanobis distance can improve diagnostic accuracy. However, when dealing with large amounts of data, it is common to use a variety of multivariate analysis tools to examine different spectral data. Linear discriminant analysis (LDA) and partial least squares (PLS) are more widely used in the application of Raman spectroscopy in gastric cancer. The idea of the LDA algorithm is to make the data as compact as possible for the same class and as dispersed as possible for different classes after projecting them into a low-dimensional space. PLS algorithm is used to extract the features of the sample data by linearly transforming the combined information of the dependent and independent variables. Other algorithms that have been used include the classification and regression tree (CART), characteristic ratio method (CRM), quadratic discriminant analysis (QDA), multinomial logistic regression (MNLR), and artificial neural network (ANN) (Table 1).

Internal and external validation, as well as sufficient supporting data, are required to improve the diagnostic model’s stability. Internal validation is the repeated training of a model using a portion of the dataset as a test set, primarily using cross-validation (e.g., leave-one-out cross validation). The use of a newly measured dataset to test the constructed model is known as external validation. The results obtained may be overly optimistic in the absence of external validation.

Machine learning and deep learning classification methods have been used to diagnose tumors from Raman spectra. Machine learning analysis methods, such as K-nearest neighbor (KNN) and support vector machine (SVM), as well as deep learning algorithms, such as ANN, convolutional neural network (CNN) (Yan et al., 2020), and self-encoder network, have wide applications in the diagnosis of other cancers (Ralbovsky and Lednev, 2020), in addition to the methods used in existing Raman studies of gastric cancer. SVM is a method to obtain data fitting models by training weight parameters, and ANN is built by simulating the human nervous system. ANN is divided into input layer, hidden layer and output layer, where the input layer imports sample data, the hidden layer describes the sample layer by layer, and the output layer is the result generated by regression analysis. Deep learning is developed based on ANN and has the advantage of handling big data and end-to-end processing. CNN, in particular, is less reliant on preprocessing (Acquarelli et al., 2017). The interpretability and reproducibility of deep learning methods, on the other hand, are important limiting factors in the increased application of spectral analysis (Yang et al., 2019).

## Conclusion and Future Perspectives

Given that current gastric cancer detection methods have many limitations, there is an urgent need to develop new techniques that can rapidly diagnose gastric cancer. Raman spectroscopy is a new method for label-free gastric cancer diagnosis that can identify the differences in chemical composition between different types of gastric cancer samples. By analyzing the molecular composition and structural differences of substances in isolated tissue or body fluid samples, Raman spectroscopy distinguishes cancerous from normal samples at the molecular level, thus enabling the early diagnosis of gastric cancer. The integration of a Raman fiber optic probe and endoscope enables real-time *in vivo* detection to help diagnose the extent and site of cancer development. Improvements in Raman spectrometer equipment and the new methods emerging from Raman data analysis are also incrementally improving the efficiency and accuracy of Raman spectroscopy in diagnosing gastric cancer.

Although Raman spectroscopy has made great progress in the diagnosis of gastric cancer, there are still many problems. How to establish a database and evaluation system for Raman spectra related to gastric cancer, how to eliminate the spectral differences caused by different spectrometers, and how to obtain higher quality Raman spectra are all challenges that need to be overcome. First, how to obtain higher-quality Raman spectra more quickly during the collection of Raman spectral data is still a problem that needs to be investigated. Due to the signal background and the stability of the laser, there are differences in the spectra measured by different Raman spectrometers. In addition, the acquisition conditions, such as the laser wavelength, laser power, and integration time, as well as different sample processing methods, will affect the signal during Raman signal collection. Therefore, it is an important research direction to explore the data acquisition methods, establish a standard sampling process, and achieve detection repeatability and stability.

In terms of the analysis methods for Raman data, the existing data analysis methods are somewhat inadequate in finding the variability of Raman peaks, and more accurate and stable data processing methods should be found to cope with large or massive amounts of spectral data. Computer science and technology, such as deep learning, has great advantages in processing Raman spectral data and Raman imaging data. These technologies may even replace pathologists in the diagnosis of gastric cancer in the future.

Finally, although Raman spectroscopy is highly accurate in identifying the differences between gastric cancer and normal tissues, this accuracy is not sufficient to detect the benignity and malignancy of tumors for the accurate diagnosis of patients’ tumors, so it is necessary to explore more deeply how to use Raman spectroscopy for the diagnosis of subtypes and stages of gastric cancer. The combination of fiber optics and endoscopy is an important direction for *in situ* real-time tumor diagnosis and is a novel and rapid method for tumor diagnosis. Although many existing studies have started to use Raman endoscopy for *in vivo* detection, more *in vitro* testing is needed for clinical application.

In conclusion, the Raman spectroscopy-based method has broad application prospects in gastric cancer diagnosis, and its application as a mature detection method in clinical diagnosis is imminent.
